# CXCR6 expression in non-small cell lung carcinoma supports metastatic process via modulating metalloproteinases

**DOI:** 10.18632/oncotarget.3194

**Published:** 2015-04-07

**Authors:** Hina Mir, Rajesh Singh, Goetz H. Kloecker, James W. Lillard, Shailesh Singh

**Affiliations:** ^1^ Department of Microbiology, Biochemistry and Immunology, Morehouse School of Medicine, Atlanta, GA, USA; ^2^ James Graham Brown Cancer Center, University of Louisville School of Medicine, Louisville, KY, USA

**Keywords:** CXCR6, CXCL16, lung cancer

## Abstract

Lung cancer (LuCa) is the leading cause of cancer-related deaths worldwide regardless of the gender. High mortality associated with LuCa is due to metastasis, molecular mechanisms of which are yet to be defined. Here, we present evidence that chemokine receptor CXCR6 and its only natural ligand, CXCL16, are significantly expressed by non-small cell lung cancer (NSCLC) and are involved in the pathobiology of LuCa. CXCR6 expression was significantly higher in two subtypes of NSCLC (adenocarcinomas-ACs and squamous cell carcinoma-SCCs) as compared to non-neoplastic tissue. Additionally, serum CXCL16 was significantly elevated in LuCa cases as compared to healthy controls. Similar to CXCR6 tissue expression, serum level of CXCL16 in AC patients was significantly higher than SCC patients. Biological significance of this axis was validated using SCC and AC cell lines. Expression of CXCR6 was higher in AC cells, which also showed higher migratory and invasive potential than SCC. Differences in migratory and invasive potential between AC and SCC were due to differential expression of metalloproteinases following CXCL16 stimulation. Hence, our findings suggest clinical and biological significance of CXCR6/CXCL16 axis in LuCa, which could be used as potential prognostic marker and therapeutic target.

## INTRODUCTION

Lung cancer (LuCa) is one of the leading causes of cancer-related deaths and is a major disease burden worldwide [1]. Non-small cell lung cancer (NSCLC) and small cell lung cancer (SCLC) are two major types of LuCa and NSCLC accounts for more than 80% of LuCa [2]. NSCLC frequently shows a preference for the regional lymph node, liver, contralateral lung, brain and bone marrow and, it is the most commonly diagnosed subtype and the major killer in Asian and Western populations [3, 4]. Most LuCa related deaths are due to the lack of effective treatment options for advanced and metastatic disease [2]. Hence, inhibition of metastasis by targeting the molecules involved in this process will be essential for reducing LuCa related mortality [5]. Unfortunately, molecular mechanisms involved in LuCa metastasis are not well defined. However, studies have shown that chemokines and their corresponding receptors play significant role in mediating the metastatic process in many cancers [6–20], including LuCa [8, 21, 22].

Chemokines are low (8–10 kDa) molecular weight chemotactic cytokines that were first shown to mediate leukocyte immune cell trafficking and protect the host from infection [23]. Immune cells bearing specific chemokine receptors, upon infection or injury, are directed to the site of insult in response to secreted chemokines [23, 24]. Cancer cells imitate this process during migration and invasion, whereby they exploit chemokine-chemokine receptor interaction to navigate to future homing or metastatic sites [6–8]. Among all known chemokines, CXCR4/CXCL12 has been widely studied and shown to be involved in pathogenesis of several cancers, including LuCa [21, 22]. However, expression and involvement of CXCR4, CCR7, CCR9, CXCR5 and CX3CR1 in various cancers reported by our laboratory and others suggest that the multi-step process of tumor progression and metastasis is regulated by multiple chemokines and their corresponding receptors [7, 8, 10–12, 16–18, 20].

In the present study, we provide evidence that CXCR6 and its natural ligand CXCL16 are highly expressed in tissue and serum of adenocarcinoma (AC) and squamous cell carcinoma (SCC) patients compared to control subjects. Using comparable cell lines derived from patients diagnosed with AC and SCC, we have shown CXCR6 is highly expressed by these cells and stimulation of CXCR6 with CXCL16 mediates cell migration and invasion, primarily by modulating matrix metalloproteinase (MMPs).

## RESULTS

### Clinical and biological significance of CXCR6 and CXCL16 in lung cancer

**Figure 1:**
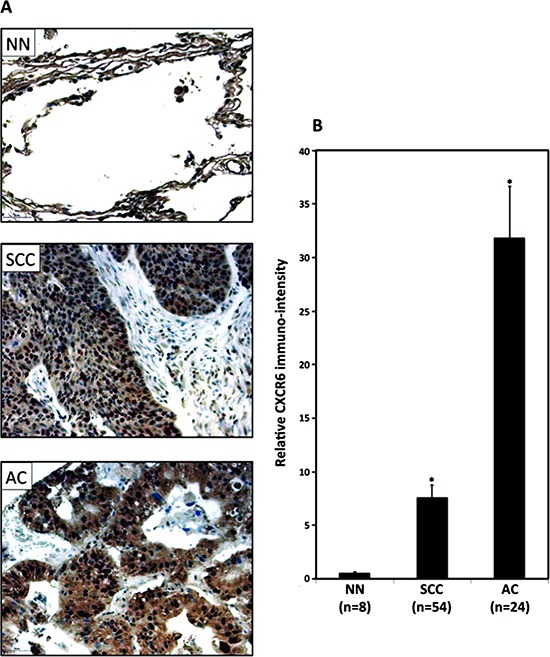
CXCR6 expression in tissues samples from LuCa patients **(A)** Representative images for CXCR6 tissue expression in lung tissues from non-neoplastic (*n* = 8), adenocarcinoma (*n* = 54) and squamous cell carcinoma (*n* = 24) were stained with isotype control or anti-CXCR6 antibodies. Brown (DAB) color shows CXCR6 staining. Images were captured using tissuefaxs cell analysis system from Tissuegnostics. **(B)** Immuno-intensities of CXCR6 were quantified using image analysis Aperio ImageScope v.6.25 software using ImageScope algorithm. ***p ≤ 0.0001 when compared between groups.

**Figure 2:**
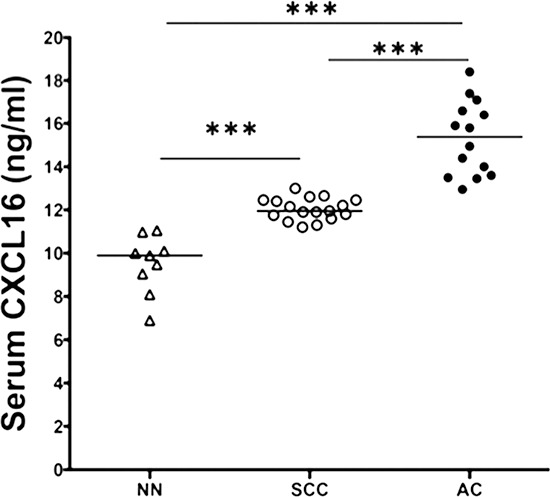
Serum CXCL16 levels in LuCa patients ELISA assays were performed to quantify CXCL16 levels in serum from patients diagnosed with (•) adenocarcinoma (*n* = 14), (○) squamous cell carcinoma (*n* = 17) and (Δ) normal healthy donors (*n* = 9). ***p ≤ 0.0001 when compared between any two groups.

Tissue microarray consisting of lung tissues from healthy individuals (non-neoplastic; *n* = 8) and LuCa patients (AC; *n* = 54 and SCC; *n* = 24) was stained for CXCR6 and corresponding immuno-intensity was analyzed. Significantly higher CXCR6 expression was found in NSCLC (AC and SCC) as compared to non-neoplastic tissues (Figure 1A and 1B). Expression of CXCR6 was significantly (*p* ≤ 0.0001) higher in AC compared to SCC. Furthermore, there was a notable difference in the CXCR6 distribution pattern between AC and SCC. Expression of CXCR6 in SCC was predominantly nuclear. However in AC, CXCR6 was largely present in cell cytoplasm and membranes, in addition to nucleus. Serum analysis revealed elevated CXCL16 levels in LuCa patients as compared to healthy individuals (Figure 2). Serum CXCL16 was significantly higher in AC (*p* ≤ 0.0001) followed by SCC compared to healthy donors (*p* ≤ 0.0001). These results suggest clinical significance of CXCR6 and CXCL16 in LuCa.

**Figure 3:**
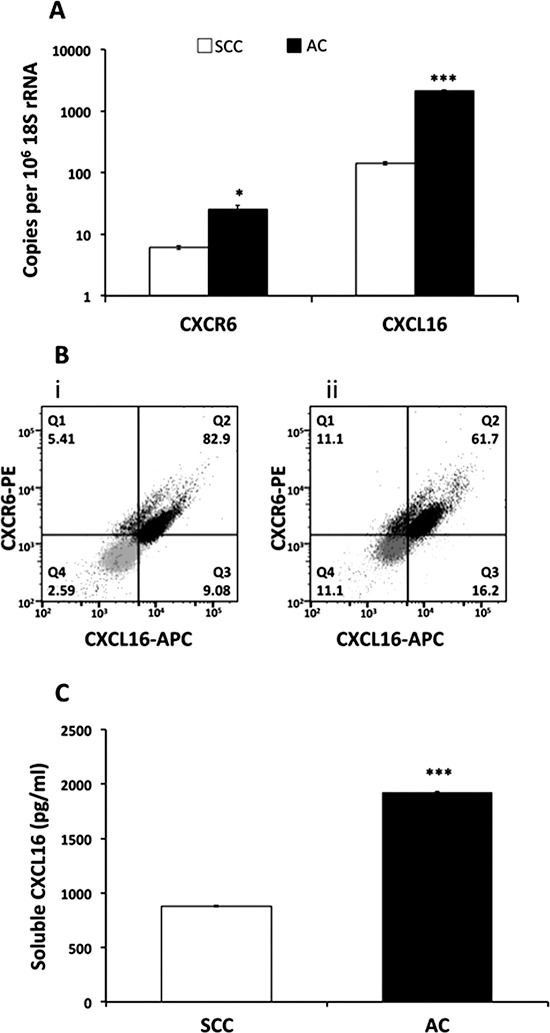
CXCR6 and CXCL16 expression in LuCa cell lines **(A)** mRNA levels by semiquantitative RT-PCR. The copies of CXCR6 and CXCL16 transcripts are expressed relative to copies of 18S rRNA. Values are mean ± SEM from 3 independent experiments. *p ≤ 0.05, ***p ≤ 0.001 as compared to SCC (NCI-H520). **(B)** Flow cytometry analysis of CXCR6 and trans-membrane CXCL16 in (i) - SCC (NCI-H520) cells and (ii) - AC (NCI-H2126) cells. Grey dots represent isotype controls for PE and APC antibody and black dots represent CXCR6-PE and CXCL16-APC in SCC (NCI-H520) and AC (NCI-H2126). CXCR6 and CXCL16 both are expressed on ~82.9% (Q2) SCC cells; 5.41% (Q1) express only CXCR6 and only CXCL16 is expressed by 9.08% (Q3) of SCC cells. In AC cells, 61.7% of the population express both receptor and ligand (Q2) whereas, 11.1 (Q1) and 16.2% (Q3) cells express only CXCR6 and CXCL16, respectively. **c)** Levels of soluble CXCL16 in LuCa supernatant. Values are mean ± SEM from 3 independent experiments. ***p ≤ 0.0001 compared to NCI-H520.

To address the biology behind altered expression of this receptor, we analyzed mRNA and protein levels of CXCR6 and CXCL16 in LuCa cell lines derived from AC (NCI-H2126) and SCC (NCI-H520) patients. Expression of CXCR6 mRNA was significantly (*p* ≤ 0.05) higher in AC as compared to SCC (Figure 3A). Similarly, FACS analysis showed higher protein expression of CXCR6 in AC as compared to SCC (Figure 3B).

Higher levels of basal CXCL16 mRNA (*p* ≤ 0.001) (Figure 3A) and soluble CXCL16 (*p* ≤ 0.0001) (Figure 3C) in AC than SCC cell lines further substantiated serum data. Levels of soluble CXCL16 in conditioned medium collected from AC was two fold higher than that from SCC cells. Flow cytometry analysis revealed variations in surface CXCL16 expression among the two cell lines (Figure 3B). The percentage of AC cells expressing only CXCL16 or CXCR6 was ~16 and 11% respectively, while ~62% AC cells expressed both CXCL16 as well as CXCR6. Interestingly, the percentage of SCC (~83%) expressing both CXCL16 and CXCR6 was higher than AC, whereas the proportion of SCC expressing only CXCL16 (~9%) or only CXCR6 (~5%) was less.

### Soluble CXCL16 induces lung cancer cell proliferation, migration and invasion

**Figure 4:**
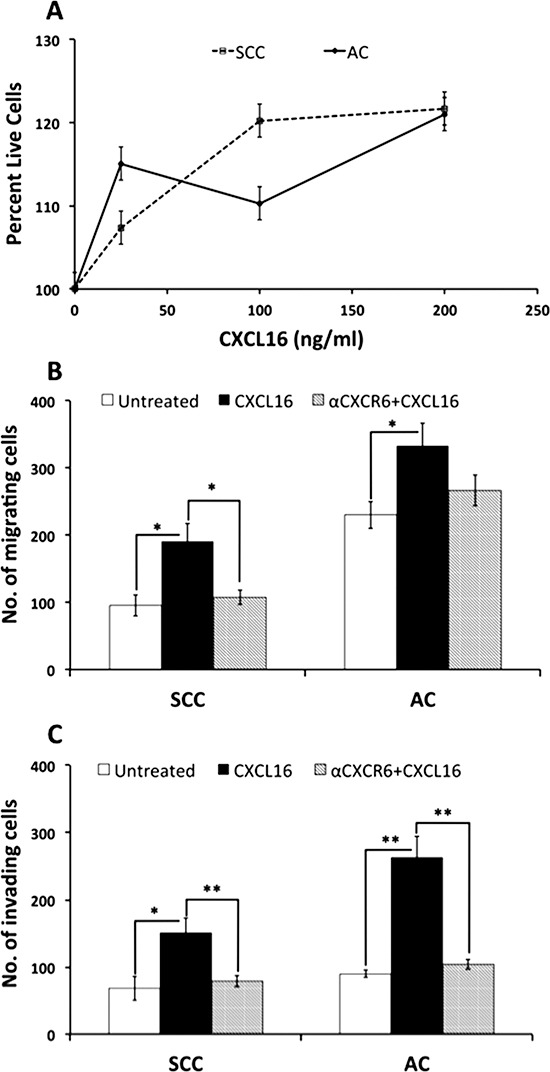
Proliferation and metastatic potential of LuCa cells with CXCL16 stimulation **(A)** LuCa cells were incubated with different concentrations of CXCL16 and cell viability was measured using MTT. Line graph represents percentage of live cells after CXCL16 addition with reference to cells without CXCL16 addition. Dashed line represents SCC (NCI-H520) cell viability and solid line indicates AC (NCI-H2126) cell viability. LuCa cells were tested for their ability to **(B)** migrate and **(C)** invade under CXCL16 chemotactic gradient in the presence and absence of anti-CXCR6 Ab (1 ug/ml). Values are mean ± SEM from 3 independent experiments. *p ≤ 0.05; **p ≤ 0.01 when compared between two indicated groups.

Chemokines have the ability to influence cellular growth and their invasive potential. Studies have shown chemokines like CXCL13 [13] and CXCL16 [14, 25] independently enhance cell proliferation as well as invasive capacity of prostate cancer cells and human trophoblast cells. High soluble CXCL16 (sCXCL16) levels, result of shedding activities like that of ADAM-10 [26], led us to study impact of sCXCL16 on the two cell types with respect to cancer progression. LuCa cells, when treated with different concentrations of recombinant CXCL16, showed more proliferation compared to untreated cells (Figure 4A).

Both cell lines were also highly responsive towards CXCL16 gradients in both migration and invasion assays (Figure 4B and 4C). Importantly, the CXCL16 gradient was created using ~100 fold higher CXCL16 concentrations than those secreted by cell lines to effectively measure this potential effect. The chemo-attractant effect of CXCL16 was specific to CXCR6 as it was inhibited in presence of anti-CXCR6 antibody.

### CXCR6/CXCL16 modulates MMP and TIMP expression in NSCLC cell lines

**Figure 5:**
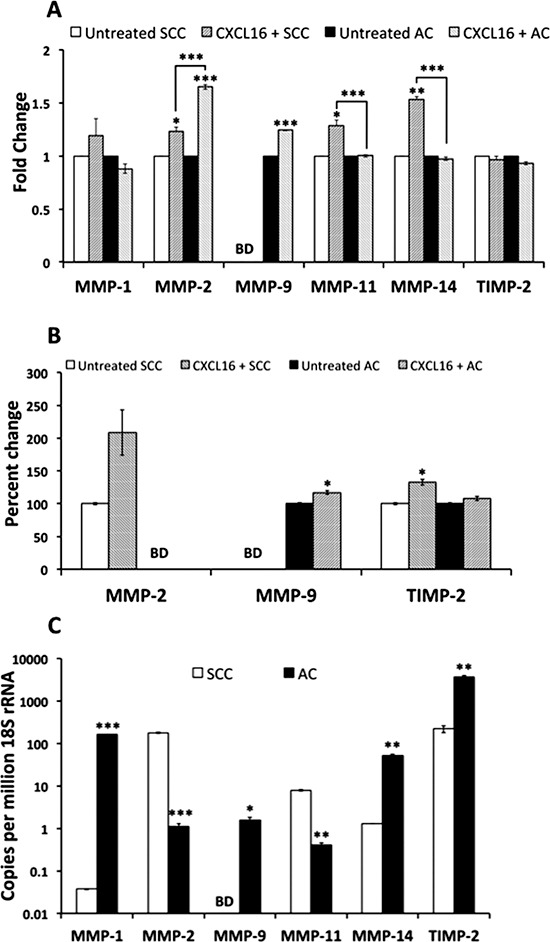
Expression of MMP and TIMP in LuCa cells **(A)** Changes in mRNA levels of MMPs and TIMP after treatment with 100 ng/ml CXCL16 for 30 minutes. Values are mean ± SEM from 3 independent experiments. *p ≤ 0.05; **p ≤ 0.01; ***p ≤ 0.001 compared to respective untreated cells or as indicated. **(B)** Percent increase in the levels of secreted MMP-2, 9 and TIMP-2 proteins 24 h after stimulation with 100 ng/ml CXCL16. *p ≤ 0.05 compared to respective untreated cells. Levels of MMP-2 in AC (NCI-H2126) and MMP-9 in SCC (NCI-H520) were below detection limit of the kit. **(C)** Basal levels of MMP and TIMP mRNA in SCC (NCI-H520) and AC (NCI-H2126) cells. Values are mean ± SEM from 3 independent experiments. *p ≤ 0.05; **p ≤ 0.01; ***p ≤ 0.001 compared to SCC. MMP-9 mRNA could not be detected in SCC cells.

Matrix metalloproteinase (MMPs) and tissue inhibitors of metalloproteinase (TIMPs) play crucial role in dissemination and invasion of tumor cells. Studies have shown MMP-1, -2, -9, -11 and -14 are highly expressed by LuCa [27–31]. Hence, we analyzed levels of these MMPs in LuCa cell lines with respect to CXCR6-CXCL16 axis (Figure 5A–5C). MMP-2 transcripts increased in both NSCLC cell lines (AC and SCC) after CXCL16 stimulation; moreover, the increase in AC was significantly higher than in SCC. In addition to this, MMP-11 and -14 mRNAs were also elevated in SCC after CXCL16 stimulation. However, MMP-9 mRNA was undetectable in SCC while, AC showed a significant increase in MMP-9 mRNA after CXCL16 stimulation. Interestingly, MMP-1, -11, -14 mRNAs in AC did not change after CXCL16 addition. CXCL16 treatment also increased MMP-2 protein levels to ~108.6% in SCC conditioned media collected 24 h after the treatment, but it was below the detection limit in conditioned media collected from AC after CXCL16 stimulation. There was marginal increase in MMP-9 (~16.7%) levels in conditioned media from AC cells after CXCL16 stimulation but its was below the detection limit in SCC conditioned media after CXCL16 stimulation. We also analyzed expression of TIMPs in LuCa cells following CXCL16 treatment. We could not detect TIMP-1 mRNA or protein in AC and SCC cell lines used in this study. Levels of TIMP-2 mRNA remained unchanged in both cell lines (Figure 5A). Furthermore, the change in TIMP-2 protein in SCC after 24 h CXCL16 treatment was significantly higher (~32.4%) (*p* ≤ 0.05) (Figure 5B). In contrast, there was no significant change in TIMP-2 protein in AC cells after CXCL16 treatment.

### ADAM 10 expression and association with soluble CXCL16 levels in NSCLC

**Figure 6:**
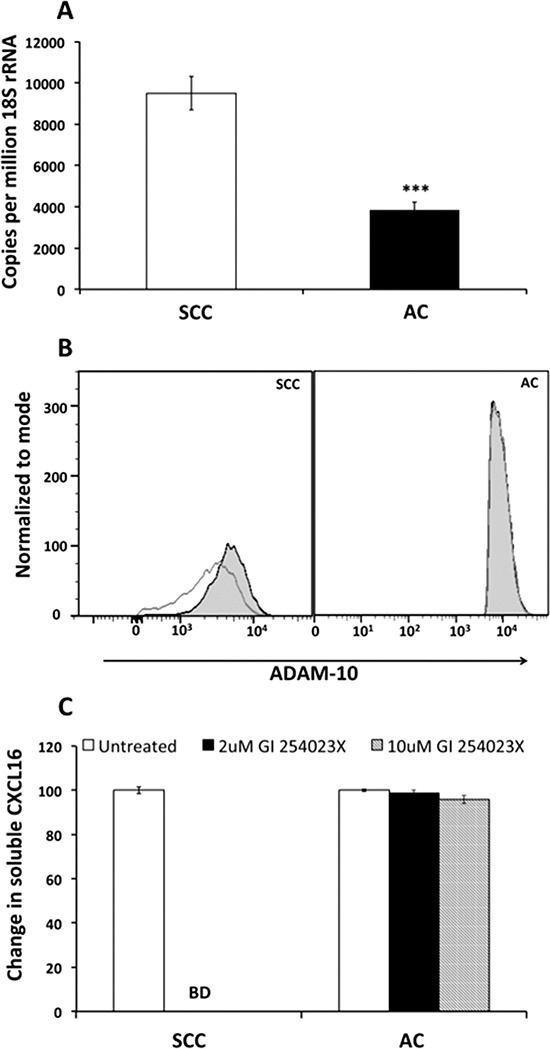
ADAM 10 expression after CXCL16 stimulation and release of CXCL16 after ADAM 10 inhibition in LuCa cells **(A)** ADAM 10 mRNA levels by semiquantitative RT-PCR. Copies of transcripts are expressed relative to copies of 18S rRNA. Values are mean ± SEM from 3 independent experiments. ***p ≤ 0.001 compared to SCC (NCI-H520). **(B)** Protein expression of ADAM 10 after CXCL16 stimulation by flow cytometry. Grey empty histograms represent untreated controls and black filled histograms represent CXCL16 treated sample in SCC (NCI-H520) and AC (NCI-H2126). **(C)** CXCL16 released after inhibition of ADAM 10 with two different concentrations of GI254023X. CXCL16 levels reduced below detection limit of the kit after inhibition of ADAM 10 in SCC (NCI-H520) cells.

Metalloproteinases are known to regulate metastatic processes [32]. Elevated ADAM 10 expression by tumors is shown to be poor prognostic indicator in many cancers [33–37]. It also plays important role in shedding of CXCL16. Interestingly, we found higher levels of ADAM 10 mRNA in SCC as compared to AC cells (Figure 6A). However, ADAM 10 protein was higher in AC cells, than SCC cells (Figure 6B). Soluble CXCL16 levels were significantly reduced in SCC culture supernatants after inhibition of ADAM 10 with its pharmacological inhibitor (GI254023X) (Figure 6C). On the other hand, soluble CXCL16 induced ADAM 10 expression in SCC cells (Figure 6B). However, such interdependence between soluble CXCL16 and ADAM 10 was not observed in AC cells (Figure 6B and 6C).

## DISCUSSION

Chemokines represent a family of small-molecular weight chemotactic cytokines with homologous structure and similar function, which bind to G protein-coupled receptors [38]. Chemokines and their corresponding receptors were primarily associated with leukocyte trafficking in the immune system [38]. Recent studies in cancer suggest their role beyond leukocyte trafficking, since cancer cells use similar mechanisms during disease progression and metastasis [39–42]. Indeed, NSCLC highly express CXCR4 and blockade of this receptor leads to inhibition of metastasis [16, 17, 20, 43–48] implying the importance of chemokine/chemokine receptor and their interaction in NSCLC metastasis. These previous studies encouraged us to examine the involvement of other chemokines in LuCa.

Interaction of CXCR6-CXCL16 regulates thymocyte development, effector T cell trafficking and promotes dendritic cells and CD8^+^T cell interactions [49]. However, dysregulation of the CXCR6/CXCL16 axis has been implicated in inflammatory conditions such as atherosclerosis and rheumatoid arthritis [50–53]. Cancer development is associated with inflammation [54] and hence involvement of this chemokine in cancer would not be surprising. Furthermore, bronchial epithelial cells express high levels of CXCL16 [55] implying that these cells are under constant challenge with CXCL16.

Our results show increased CXCR6/CXCL16 expression in NSCLC proposing a role of this dysregulated axis in LuCa progression and metastasis. Owing to their origin from glandular epithelial cells, ACs are more aggressive in nature and relatively less responsive to chemotherapy. Significantly higher expression of CXCR6/CXCL16 in AC patient samples and cell lines could be involved in maintaining or dictating the aggressive phenotype of AC over SCC. Besides, nuclear localization of CXCR6, typically a cell membrane protein, in SCC tissues could also be of significance. Reports from our and other labs have shown nuclear localization of CXCR5 [16] and CXCR4 [21, 56] in prostate and, breast and lung cancer respectively. These nuclear localized CXCR4 are functional and underlie the recurrence of prostate cancer [57]. Similar role could be played by CXCR6 in SCC tissues with respect to LuCa, however further in-depth studies are required to conclude this facet.

CXCL16 has been assigned certain roles depending on its state. Trans-membrane CXCL16 promotes cell-cell adhesion [58], lymphocyte accumulation at tumor sites and leads to tumor immunity and better prognosis [59]. In contrast, soluble CXCL16 is pro-mitogenic, antiapoptotic and correlates with increased metastasis and poor prognosis [59, 60]. Hence, results showing significantly high levels of soluble CXCL16, i.e. in serum or conditioned media of NSCLC are particularly important with respect to the development of LuCa.

Cell lines derived from AC, with proportionally higher expression of CXCR6, show greater migratory and invasive potential than SCC cell lines under the chemotactic gradient of CXCL16. Here, LuCa cells expressing only CXCR6 and no trans-membrane CXCL16 would be more responsive to the elevated CXCL16 than those expressing both the membrane bound ligand and the receptor. Blocking CXCR6 with antibody significantly reduced the migration and invasion of SCC cells; however, only invasion was affected in AC cells. It should be noted here that invasion, as assessed using collagen IV matrix, is mainly attributed to the activity of MMP-2 and -9, the collagenases affected differentially by CXCL16 in the two cell lines; whereas migration is associated with a different set of proteins involved in motility.

Recombinant CXCL16 significantly increased proliferation in both AC and SCC derived cell lines substantiating its pro-mitogenic role. In other words, the observed elevated serum CXCL16 levels may favor neoplastic transformation during LuCa via promoting migration and invasion along with enhancing proliferation of cells. Concomitantly, soluble CXCL16 contributed by LuCa cells could saturate CXCR6 on lymphocytes and block their ability to detect cancer by mimicking trans-membrane CXCL16. It is also noteworthy that membrane CXCL16 is expressed by a large number of organs including brain, kidney and colon during inflammation [61–63] and this could be the cause of wider metastatic array of LuCa once the cells have dislodged from the original site of tumor.

Cleavage of CXCL16 is an event associated with protease activity of ADAM 10 in many cancers [26]. Decrease in CXCL16 release by SCC cells after ADAM 10 inhibition and induction of ADAM 10 expression by sCXCL16 implies a positive feed forward mechanism between these two proteins in SCC. A study shows CXCL16 induces upregulation of TNF-α, a poor prognostic marker associated with NSCLC [64, 65], in NF-kappa B dependent manner [60]. A concurrent study demonstrates TNF-α induced CXCL16 mRNA expression in endothelial cells [26], while ADAM 10 is associated with increased bioavailability/shedding of TNF-α [66–68]. Hence, it is likely that in SCC as well ADAM 10 is involved in this CXCL16 and TNF-α loop. Unlike SCC cells, AC cells may have an alternate mechanism to control membrane-bound versus soluble CXCL16 since neither ADAM 10 and soluble CXCL16 were proportional to each other nor did ADAM 10 inhibition affect CXCL16 release in these cells.

In addition to ADAM 10, we show significant changes in MMP -2, -9, -11, -14 and TIMP-2 expressions in LuCa cells, after CXCL16 treatment. MMP-2 and MMP-9 cleave type IV collagen, the major structural protein for ECM and basement membrane and are consequently associated with tumor development [32, 69, 70]. Stromelysin-3 or MMP-11 is implicated in extracellular matrix remodeling [71], increased tumor uptake [72] along with anti apoptotic and anti necrotic effect during cancer progression [73, 74]. MMP-14 and TIMP-2 are required for activation of pro-MMP-2 [75]. MMPs and their inhibitor, TIMPs, are commonly linked to metastasis of neoplastic cells, but these proteases have other more specific roles in cancer. Increased TIMP-2 could function by modulating I*κ*B*α* and IL-8 levels, phosphorylation status of I*κ*B*α* and NF-*κ*B [76] and thereby directly influence lung cancer progression. In all, induction of these MMPs and TIMP-2 after stimulation with recombinant CXCL16 clearly implicates its role in promoting LuCa metastasis.

The difference in expression patterns of MMPs between two cell lines after CXCL16 stimulation is not unusual and could be due to several reasons. For example, prostate cancer cell lines show variations in their MMP profile post CXCL16 addition [14]. Analysis of basal expression, i.e., in absence of additional CXCL16, shows that the two cell lines do not express same type or concentration of MMPs. Moreover, the fact that both cell lines were derived from different milieu [77], absence of stromal microenvironment [78], and differential CXCR6 expression altogether could influence MMPs. Nonetheless, differential effect of CXCL16 on MMPs possibly resulting in variations in migration and invasion capacity of cells suggests differences in mechanisms of tumor progression exploited by the two cell types.

## METHODS

### Immunohistochemistry and quantitation

LuCa tissue microarray (TMA) slides were obtained from Abxis. Lung cancer TMA consisting of Non-neoplastic (*n* = 8), adenocarcinoma (*n* = 54) and squamous cell carcinoma (*n* = 24) were de-paraffinized in xylene and rehydrated through a graded series of ethanol (100%, 95% and 70%) for 5 min in each series and washed in distilled water. Endogenous peroxidase activity was blocked by incubating with 3% H_2_O_2_ in PBS for 5 min. The slides were then rinsed 3 times with deionized water followed by three washes in Tris-buffer and incubated with Fc block (Innovex Bioscience) for 30 min at RT in a humidified chamber. To reduce non-specific binding, the sections were then washed with Tris-buffer and incubated with 3% normal goat serum for 1 h at RT. Unbound goat serum was removed with Tris-buffer, and the sections were incubated with 5.0 μg/ml anti-CXCR6 antibody (MAB699, R&D Systems) for 90 min in humidified chamber at RT. Negative control slide was incubated with 5.0 μg/ml mouse isotype control antibody (R&D Systems). The sections were then washed with Tris-buffer and incubated with Horseradish peroxidase (HRP)-conjugated goat anti-mouse antibody (Zymed) for 20 min at RT. After incubation, sections were washed and incubated with DAB at RT until color developed. Next, the slides were washed in deionized H_2_O and counter-stained with Mayer's hematoxylin (Sigma) for 1 min. Subsequently, sections were washed with water, dehydrated in 70%, 95% and absolute alcohol for 5 min each and passed through xylene 3 times for 1 min each; and finally mounted with permount (Sigma).

To numerically analyze the immunohistochemical staining, virtual slides were created from stained samples after scanning each specimen using an Aperio ScanScope scanning system (Aperio Technologies). A color markup image for each slide was obtained based on membrane staining. The ScanScope generated true color digital images of each stained sample, which were viewed using Aperio ImageScope version 6.25 software. The ImageScope algorithm was used to determine staining intensity for each sample by digitally analyzing the color intensity.

### Cell lines and cell culture

Non-small cell lung cancer (NSCLC) cell lines, NCI-H520 (HTB-182; derived from a SCC patient) and NCI-H2126 (CCL-256; derived from an AC patient) were obtained from ATCC. NCI-H520 cells were cultured in RPMI-1640 supplemented with 10% fetal bovine serum (HyClone). NCI-H2126 cells were cultured in HITES medium i.e. DMEM: F12 medium supplemented with 0.005 mg/ml Insulin (Sigma), 0.01 mg/ml Transferrin (Sigma), 30 nM Sodium selenite (Sigma), 10 nM Hydrocortisone (Sigma), 10 nM beta-estradiol (Sigma), extra 2 mM L-glutamine (Hyclone) along with 5% fetal bovine serum. Both the cell lines were maintained in a 37°C incubator with 5% CO_2_.

### RNA isolation and RT-qPCR analysis

Total RNA was isolated from CXCL16-treated and untreated LuCa cells using Tri-Reagent (Sigma). RNA was precipitated and resuspended in RNA Secure (Ambion). Complementary DNA was generated by reverse transcribing 1.0 μg of total RNA using Verso cDNA Synthesis kit (Thermo Scientific) using random hexamers according to manufacturer's protocols. mRNA levels of CXCR6, CXCL16, MMP-1, MMP-2, MMP-9, MMP-11, MMP-14, TIMP-2, ADAM-10 and 18S rRNA were determined using gene specific primers with iQ SYBR-Green Supermix (Bio-Rad, CA). The real-time thermal cycler (CFX96 Touch, Bio-Rad) profile used for amplification was as follows: initial denaturation 95°C for 3 min; denaturation 95°C for 10 sec; and annealing, extension, and detection at 60°C for 45 sec for 40 cycles. mRNA expression of the aforementioned targets is represented relative to 18S rRNA or fold change in expression relative to controls. Gene expression experiments were repeated three times to validate the results.

### Flow cytometry

LuCa cells (1 × 10^6^) were washed thrice with fluorescence-activated cell-sorting (FACS) buffer (PBS supplemented with 2% fetal bovine serum). Cells were then stained with respective antibodies as per manufacturer's instruction at 4°C for 40 min. Subsequently cells were washed twice with FACS buffer to remove unbound antibodies. Next, labeled cells were fixed in 500 μL of 2% paraformaldehyde solution, washed and analyzed by flow cytometry using a FACS ARIA-II flow cytometer (BD Biosciences, CA) and flow Jo 10.0.6 software (Treestar Inc). PE-conjugated mouse anti-human CXCR6 (FAB699P) antibody, PE-conjugated mouse anti-human ADAM-10 (IC1427P) antibody, APC-conjugated rat anti-human CXCL16 (FAD976A) antibody, PE-conjugated mouse IgG2B immunoglobulin isotype control (IC0041P) and APC-conjugated rat IgG2A immunoglobulin isotype control (IC006A) were purchased from R&D Systems (Minneapolis, MN).

### ELISA

Sera from patients diagnosed with SCC (*n* = 17) or AC (*n* = 14), and from healthy controls (*n* = 9), were provided by Dr. Goetz H. Kloecker of the James Graham Brown Cancer Center, University of Louisville, Louisville, KY. Healthy donors had no active lung disease or symptoms at the time of blood collection. All subjects gave written informed consent. The University of Louisville IRB approved the use of these diagnostic specimens in accordance with the Department of Health and Human Service Policy for the Protection of Human Research Subjects 45 CFR 46.101(b) 2 and use of archived de-identified materials. Serum CXCL16 levels were quantified by human CXCL16 quantikine ELISA kit (R&D Systems) according to the manufacturer's protocol. Briefly, 100 μl of assay diluent (provided with the kit), followed by 50 μl of standards, controls, and serum samples, were added in different wells of an ELISA plate and incubated for 2 h at RT. Following washing four times with quantikine wash buffer 1 (provided with the kit), 200 μl of conjugate (antibody) was added to each well, and the plate was further incubated for 2 h at RT. The plate was washed, 200 μl of substrate solution was added, and the plate was incubated for 30 min in the dark at RT. After incubation, 50 μl of stop solution (2N H_2_SO_4_) was added to each well, and the optical density was measured with a microplate ELISA reader at 450 nm with the wavelength correction set at 540 nm. Each sample was tested in duplicate for assessment of inter-assay variation.

For measuring CXCL16 in culture supernatants, LuCa cells (0.5 × 10^6^/well) were seeded in six well plates and conditioned medium was collected after 24 h for CXCL16 ELISA. MMP-2, MMP-9, TIMP-1 and TIMP-2 protein levels were measured from conditioned medium collected 24 h after addition of recombinant CXCL16 (100 ng/ml). Commercially available ELISA kits (R&D systems) were used for the assays.

### Cell proliferation assay

LuCa cells were seeded at a density of 2 × 10^4^/100 ul/well in 96 well plates. Next day, keeping the volume constant, cells were stimulated with different concentration (0–200 ng/ml) of recombinant CXCL16 (Peprotech). After 24 h, cell proliferation was assessed using MTT assay [79]. Briefly, MTT (20 μL of 5 mg/ml in PBS) was added in each well and plate was incubated at 37°C for 3 h. Resultant formazan crystals were dissolved in 100 μL of DMSO and absorbance was recorded in a spectrophotometer with plate reader at 570 nm. All concentrations were tested in triplicates and the experiment was repeated three times.

### Migration and invasion assays

Migration and invasion studies were performed using BD Biocoat migration and Matrigel invasion chambers (Becton-Dickson Labware), respectively. Serum free DMEM was added to bottom and top chamber of inserts and allowed to hydrate for 2 h at 37°C with 5% CO_2_. Next, 0.5 × 10^5^ cells were added to the top chamber of inserts and 100 ng/ml CXCL16 (Peprotech, NJ) was added as chemo-attractant in the bottom chamber. To determine if the migration and invasion of LuCa cells is mediated specifically by CXCR6-CXCL16 interaction, cells pre-incubated with 1.0 μg/ml anti-CXCR6 antibody (MAB699, R&D Systems) were added to the top chamber in one well of Matrigel or control inserts and allowed to migrate/invade under chemotactic gradient of CXCL16 for overnight at 37°C and 5% CO_2_. After incubation, non-migrating cells on the upper surface of the membrane were removed with a cotton swab. Cells at the bottom surface of the insert were fixed with 100% methanol for 2 min, stained for 2 min with crystal violet (Fisher Scientific), and rinsed twice with de-ionized water. Migrated/invaded cells were counted by microscopy at 40× magnification. All experiments were repeated three times to validate the results.

### Statistics

Comparisons of CXCR6 expression immunointensity in lung TMA and comparison of CXCL16 levels in serum of healthy controls and NSCLC patients were made by non-parametric Mann Whitney U test. Results were declared significant at α level of 0.05. Expression of CXCR6, CXCL16, MMPs and TIMP-2 mRNA and/or protein in LuCa cell lines was compared using a two-tailed Student's t-test and expressed as means ± SEM. Results of migration and invasion assays were analyzed by one-way ANOVA. Values were declared significantly different at a level of 0.05.

## CONCLUSIONS

Our study strongly suggests that CXCR6-CXCL16 interaction plays crucial role in LuCa pathobiology. Differential expression of CXCR6/CXCL16 in two NSCLC subtypes namely SCC and AC could be correlated with their prognostic differences. Serum CXCL16 can be used as independent predictor of poor prognosis or survival in NSCLC cases with further validation in larger groups. Our results also demonstrate the biological significance of CXCR6/CXCL16 in NSCLC cell lines and that this axis could be an effective target in NSCLC. Nevertheless, carefully designed approaches would be required to target this immunologically important receptor-chemokine axis.

## Abbreviations

AC: Adenocarcinoma
SCC: Squamous cell carcinoma
LuCa: lung cancer
NSCLC: Non-small cell lung carcinoma
SCLC: Small cell lung carcinoma

## References

[1] Siegel R, Ma J, Zou Z, Jemal A (2014). Cancer statistics. CA: a cancer journal for clinicians.

[2] Wood SL, Pernemalm M, Crosbie PA, Whetton AD (2014). The role of the tumor-microenvironment in lung cancer-metastasis and its relationship to potential therapeutic targets. Cancer Treat Rev.

[3] Fidler IJ (1990). Critical factors in the biology of human cancer metastasis: twenty-eighth G.H.A. Clowes memorial award lecture. Cancer research.

[4] Fidler IJ (1999). Critical determinants of cancer metastasis: rationale for therapy. Cancer chemotherapy and pharmacology.

[5] Chemotherapy in non-small cell lung cancer (1995). a meta-analysis using updated data on individual patients from 52 randomised clinical trials. Non-small Cell Lung Cancer Collaborative Group. BMJ (Clinical research ed).

[6] Burger JA, Kipps TJ (2006). CXCR4: a key receptor in the crosstalk between tumor cells and their microenvironment. Blood.

[7] Darash-Yahana M, Pikarsky E, Abramovitch R, Zeira E, Pal B, Karplus R, Beider K, Avniel S, Kasem S, Galun E, Peled A (2004). Role of high expression levels of CXCR4 in tumor growth, vascularization, and metastasis. FASEB Journal.

[8] Kakinuma T, Hwang ST (2006). Chemokines, chemokine receptors, and cancer metastasis. Journal of leukocyte biology.

[9] Matsumura S, Demaria S (2010). Up-regulation of the pro-inflammatory chemokine CXCL16 is a common response of tumor cells to ionizing radiation. Radiation Research.

[10] Muller A, Homey B, Soto H, Ge N, Catron D, Buchanan ME, McClanahan T, Murphy E, Yuan W, Wagner SN, Barrera JL, Mohar A, Verastegui E, Zlotnik A (2001). Involvement of chemokine receptors in breast cancer metastasis. Nature.

[11] Shulby SA, Dolloff NG, Stearns ME, Meucci O, Fatatis A (2004). CX3CR1-fractalkine expression regulates cellular mechanisms involved in adhesion, migration, and survival of human prostate cancer cells. Cancer research.

[12] Zhang Z, Ni C, Chen W, Wu P, Wang Z, Yin J, Huang J, Qiu F (2014). Expression of CXCR4 and breast cancer prognosis: a systematic review and meta-analysis. BMC Cancer.

[13] El-Haibi CP, Singh R, Sharma PK, Singh S, Lillard JW (2011). CXCL13 mediates prostate cancer cell proliferation through JNK signalling and invasion through ERK activation. Cell Prolif.

[14] Hu W, Zhen X, Xiong B, Wang B, Zhang W, Zhou W (2008). CXCR6 is expressed in human prostate cancer in vivo and is involved in the in vitro invasion of PC3 and LNCap cells. Cancer Science.

[15] Salazar N, Castellan M, Shirodkar SS, Lokeshwar BL (2013). Chemokines and chemokine receptors as promoters of prostate cancer growth and progression. Crit Rev Eukaryot Gene Expr.

[16] Singh S, Singh R, Singh UP, Rai SN, Novakovic KR, Chung LW, Didier PJ, Grizzle WE, Lillard JW (2009). Clinical and biological significance of CXCR5 expressed by prostate cancer specimens and cell lines. International Journal of Cancer.

[17] Singh S, Singh UP, Stiles JK, Grizzle WE, Lillard JW (2004). Expression and functional role of CCR9 in prostate cancer cell migration and invasion. Clinical Cancer Research.

[18] Vindrieux D, Escobar P, Lazennec G (2009). Emerging roles of chemokines in prostate cancer. Endocr Relat Cancer.

[19] Gooden MJ, Wiersma VR, Boerma A, Leffers N, Boezen HM, ten Hoor KA, Hollema H, Walenkamp AM, Daemen T, Nijman HW, Bremer E (2014). Elevated serum CXCL16 is an independent predictor of poor survival in ovarian cancer and may reflect pro-metastatic ADAM protease activity. British Journal of Cancer.

[20] Singh R, Stockard CR, Grizzle WE, Lillard JW, Singh S (2011). Expression and histopathological correlation of CCR9 and CCL25 in ovarian cancer. International journal of oncology.

[21] Spano JP, Andre F, Morat L, Sabatier L, Besse B, Combadiere C, Deterre P, Martin A, Azorin J, Valeyre D, Khayat D, Le Chevalier T, Soria JC (2004). Chemokine receptor CXCR4 and early-stage non-small cell lung cancer: pattern of expression and correlation with outcome. Annals of Oncology.

[22] Wald O, Shapira OM, Izhar U (2013). CXCR4/CXCL12 axis in non small cell lung cancer (NSCLC) pathologic roles and therapeutic potential. Theranostics.

[23] Schall TJ, Bacon KB (1994). Chemokines, leukocyte trafficking, and inflammation. Current opinion in immunology.

[24] Kim CH, Broxmeyer HE (1999). Chemokines: signal lamps for trafficking of T and B cells for development and effector function. Journal of leukocyte biology.

[25] Huang Y, Zhu XY, Du MR, Wu X, Wang MY, Li DJ (2006). Chemokine CXCL16, a scavenger receptor, induces proliferation and invasion of first-trimester human trophoblast cells in an autocrine manner. Hum Reprod.

[26] Abel S, Hundhausen C, Mentlein R, Schulte A, Berkhout TA, Broadway N, Hartmann D, Sedlacek R, Dietrich S, Muetze B, Schuster B, Kallen KJ, Saftig P, Rose-John S, Ludwig A (2004). The transmembrane CXC-chemokine ligand 16 is induced by IFN-gamma and TNF-alpha and shed by the activity of the disintegrin-like metalloproteinase ADAM10. J Immunol.

[27] Sauter W, Rosenberger A, Beckmann L, Kropp S, Mittelstrass K, Timofeeva M, Wolke G, Steinwachs A, Scheiner D, Meese E, Sybrecht G, Kronenberg F, Dienemann H, Chang-Claude J, Illig T, Wichmann HE (2008). Matrix metalloproteinase 1 (MMP1) is associated with early-onset lung cancer. “Cancer Epidemiology, Biomarkers & Prevention”.

[28] Guo CB, Wang S, Deng C, Zhang DL, Wang FL, Jin XQ (2007). Relationship between matrix metalloproteinase 2 and lung cancer progression. Mol Diagn Ther.

[29] Zheng S, Chang Y, Hodges KB, Sun Y, Ma X, Xue Y, Williamson SR, Lopez-Beltran A, Montironi R, Cheng L (2010). Expression of KISS1 and MMP-9 in non-small cell lung cancer and their relations to metastasis and survival. Anticancer Res.

[30] Kren L, Goncharuk VN, Krenova Z, Stratil D, Hermanova M, Skrickova J, Sheehan CE, Ross JS (2006). Expression of matrix metalloproteinases 3, 10 and 11 (stromelysins 1, 2 and 3) and matrix metalloproteinase 7 (matrilysin) by cancer cells in non-small cell lung neoplasms. Clinicopathologic studies. Cesk Patol.

[31] Atkinson JM, Pennington CJ, Martin SW, Anikin VA, Mearns AJ, Loadman PM, Edwards DR, Gill JH (2007). Membrane type matrix metalloproteinases (MMPs) show differential expression in non-small cell lung cancer (NSCLC) compared to normal lung: correlation of MMP-14 mRNA expression and proteolytic activity. Eur J Cancer.

[32] Di Nezza LA, Misajon A, Zhang J, Jobling T, Quinn MA, Ostor AG, Nie G, Lopata A, Salamonsen LA (2002). Presence of active gelatinases in endometrial carcinoma and correlation of matrix metalloproteinase expression with increasing tumor grade and invasion. Cancer.

[33] Ko SY, Lin SC, Wong YK, Liu CJ, Chang KW, Liu TY (2007). Increase of disintergin metalloprotease 10 (ADAM10) expression in oral squamous cell carcinoma. Cancer letters.

[34] Carl-McGrath S, Lendeckel U, Ebert M, Roessner A, Rocken C (2005). The disintegrin-metalloproteinases ADAM9, ADAM12, and ADAM15 are upregulated in gastric cancer. International journal of oncology.

[35] Fogel M, Gutwein P, Mechtersheimer S, Riedle S, Stoeck A, Smirnov A, Edler L, Ben-Arie A, Huszar M, Altevogt P (2003). L1 expression as a predictor of progression and survival in patients with uterine and ovarian carcinomas. Lancet.

[36] Mechtersheimer S, Gutwein P, Agmon-Levin N, Stoeck A, Oleszewski M, Riedle S, Postina R, Fahrenholz F, Fogel M, Lemmon V, Altevogt P (2001). Ectodomain shedding of L1 adhesion molecule promotes cell migration by autocrine binding to integrins. The Journal of cell biology.

[37] Gutwein P, Oleszewski M, Mechtersheimer S, Agmon-Levin N, Krauss K, Altevogt P (2000). Role of Src kinases in the ADAM-mediated release of L1 adhesion molecule from human tumor cells. The Journal of biological chemistry.

[38] Zlotnik A, Yoshie O (2000). Chemokines: a new classification system and their role in immunity. Immunity.

[39] Perissinotto E, Cavalloni G, Leone F, Fonsato V, Mitola S, Grignani G, Surrenti N, Sangiolo D, Bussolino F, Piacibello W, Aglietta M (2005). Involvement of chemokine receptor 4/stromal cell-derived factor 1 system during osteosarcoma tumor progression. Clinical cancer research: an official journal of the American Association for Cancer Research.

[40] Zlotnik A (2004). Chemokines in neoplastic progression. Seminars in cancer biology.

[41] Horikawa T, Kaizaki Y, Kato H, Furukawa M, Yoshizaki T (2005). Expression of interleukin-8 receptor A predicts poor outcome in patients with nasopharyngeal carcinoma. The Laryngoscope.

[42] Wang JM, Deng X, Gong W, Su S (1998). Chemokines and their role in tumor growth and metastasis. Journal of immunological methods.

[43] Gupta P, Sharma PK, Mir H, Singh R, Singh N, Kloecker GH, Lillard JW, Singh S (2014). CCR9/CCL2 expression in non-small cell lung cancer correlates with aggressive disease and mediates key steps of metastasis. Oncotarget.

[44] Zhang XW, Qin X, Qin CY, Yin YL, Chen Y, Zhu HL (2013). Expression of monocyte chemoattractant protein-1 and CC chemokine receptor 2 in non-small cell lung cancer and its significance. Cancer immunology, immunotherapy: CII.

[45] Lin S, Sun L, Hu J, Wan S, Zhao R, Yuan S, Zhang L (2009). Chemokine C-X-C motif receptor 6 contributes to cell migration during hypoxia. Cancer letters.

[46] Liu J, Zhang L, Wang C (2012). CCL21 modulates the migration of NSCL cancer by changing the concentration of intracellular Ca2+. Oncology reports.

[47] Oonakahara K, Matsuyama W, Higashimoto I, Kawabata M, Arimura K, Osame M (2004). Stromal-derived factor-1alpha/CXCL12-CXCR 4 axis is involved in the dissemination of NSCLC cells into pleural space. American journal of respiratory cell and molecular biology.

[48] Phillips RJ, Burdick MD, Lutz M, Belperio JA, Keane MP, Strieter RM (2003). The stromal derived factor-1/CXCL12-CXC chemokine receptor 4 biological axis in non-small cell lung cancer metastases. American journal of respiratory and critical care medicine.

[49] Matloubian M, David A, Engel S, Ryan JE, Cyster JG (2000). A transmembrane CXC chemokine is a ligand for HIV-coreceptor Bonzo. Nature Immunology.

[50] Heydtmann M, Lalor PF, Eksteen JA, Hubscher SG, Briskin M, Adams DH (2005). CXC chemokine ligand 16 promotes integrin-mediated adhesion of liver-infiltrating lymphocytes to cholangiocytes and hepatocytes within the inflamed human liver. Journal of Immunology.

[51] Nanki T, Shimaoka T, Hayashida K, Taniguchi K, Yonehara S, Miyasaka N (2005). Pathogenic role of the CXCL16-CXCR6 pathway in rheumatoid arthritis. Arthritis & Rheumatism.

[52] Sato T, Thorlacius H, Johnston B, Staton TL, Xiang W, Littman DR, Butcher EC (2005). Role for CXCR6 in recruitment of activated CD8+ lymphocytes to inflamed liver. Journal of Immunology.

[53] van der Voort R, van Lieshout AW, Toonen LW, Sloetjes AW, van den Berg WB, Figdor CG, Radstake TR, Adema GJ (2005). Elevated CXCL16 expression by synovial macrophages recruits memory T cells into rheumatoid joints. Arthritis & Rheumatism.

[54] Lu H, Ouyang W, Huang C (2006). Inflammation, a key event in cancer development. Mol Cancer Res.

[55] Day C, Patel R, Guillen C, Wardlaw AJ (2009). The chemokine CXCL16 is highly and constitutively expressed by human bronchial epithelial cells. Experimental Lung Research.

[56] Woo SU, Bae JW, Kim HG, Choi SH, Kang DH, Lee JB, Koo BW (2007). Correlation between the in vitro ATP-based chemosensitivity assay and HER2/neu expression in women with breast cancer. J Int Med Res.

[57] Don-Salu-Hewage AS, Chan SY, McAndrews KM, Chetram MA, Dawson MR, Bethea DA, Hinton CV (2013). Cysteine (C)-x-C receptor 4 undergoes transportin 1-dependent nuclear localization and remains functional at the nucleus of metastatic prostate cancer cells. PloS one.

[58] Shimaoka T, Nakayama T, Fukumoto N, Kume N, Takahashi S, Yamaguchi J, Minami M, Hayashida K, Kita T, Ohsumi J, Yoshie O, Yonehara S (2004). Cell surface-anchored SR-PSOX/CXC chemokine ligand 16 mediates firm adhesion of CXC chemokine receptor 6-expressing cells. Journal of leukocyte biology.

[59] Deng L, Chen N, Li Y, Zheng H, Lei Q (2010). CXCR6/CXCL16 functions as a regulator in metastasis and progression of cancer. Biochimica et Biophysica Acta: Protein Structure and Molecular Enzymology.

[60] Chandrasekar B, Bysani S, Mummidi S (2004). CXCL16 signals via Gi, phosphatidylinositol 3-kinase, Akt, I kappa B kinase, and nuclear factor-kappa B and induces cell-cell adhesion and aortic smooth muscle cell proliferation. Journal of Biological Chemistry.

[61] Ludwig A, Schulte A, Schnack C, Hundhausen C, Reiss K, Brodway N, Held-Feindt J, Mentlein R (2005). Enhanced expression and shedding of the transmembrane chemokine CXCL16 by reactive astrocytes and glioma cells. Journal of Neurochemistry.

[62] Xia Y, Entman ML, Wang Y (2013). Critical role of CXCL16 in hypertensive kidney injury and fibrosis. Hypertension.

[63] Kalliomaki M, Rajala S, Elamo H, Ashorn M, Ruuska T (2014). Increased expression of CXCL16, a bacterial scavenger receptor, in the colon of children with ulcerative colitis. J Crohns Colitis.

[64] Tas F, Duranyildiz D, Argon A, Oguz H, Camlica H, Yasasever V, Topuz E (2005). Serum levels of leptin and proinflammatory cytokines in advanced-stage non-small cell lung cancer. Med Oncol.

[65] Derin D, Soydinc HO, Guney N, Tas F, Camlica H, Duranyildiz D, Yasasever V, Topuz E (2008). Serum levels of apoptosis biomarkers, survivin and TNF-alpha in nonsmall cell lung cancer. Lung Cancer.

[66] Hikita A, Tanaka N, Yamane S, Ikeda Y, Furukawa H, Tohma S, Suzuki R, Tanaka S, Mitomi H, Fukui N (2009). Involvement of a disintegrin and metalloproteinase 10 and 17 in shedding of tumor necrosis factor-alpha. Biochemistry and cell biology = Biochimie et biologie cellulaire.

[67] Lunn CA, Fan X, Dalie B, Miller K, Zavodny PJ, Narula SK, Lundell D (1997). Purification of ADAM 10 from bovine spleen as a TNFalpha convertase. FEBS letters.

[68] Mezyk-Kopec R, Bzowska M, Stalinska K, Chelmicki T, Podkalicki M, Jucha J, Kowalczyk K, Mak P, Bereta J (2009). Identification of ADAM10 as a major TNF sheddase in ADAM17-deficient fibroblasts. Cytokine.

[69] Berube M, Deschambeault A, Boucher M, Germain L, Petitclerc E, Guerin SL (2005). MMP-2 expression in uveal melanoma: differential activation status dictated by the cellular environment. Mol Vis.

[70] Sato T, Sakai T, Noguchi Y, Takita M, Hirakawa S, Ito A (2004). Tumor-stromal cell contact promotes invasion of human uterine cervical carcinoma cells by augmenting the expression and activation of stromal matrix metalloproteinases. Gynecol Oncol.

[71] Noel A, Boulay A, Kebers F, Kannan R, Hajitou A, Calberg-Bacq CM, Basset P, Rio MC, Foidart JM (2000). Demonstration in vivo that stromelysin-3 functions through its proteolytic activity. Oncogene.

[72] Noel AC, Lefebvre O, Maquoi E, VanHoorde L, Chenard MP, Mareel M, Foidart JM, Basset P, Rio MC (1996). Stromelysin-3 expression promotes tumor take in nude mice. Journal of Clinical Investigation.

[73] Boulay A, Masson R, Chenard MP, El Fahime M, Cassard L, Bellocq JP, Sautes-Fridman C, Basset P, Rio MC (2001). High cancer cell death in syngeneic tumors developed in host mice deficient for the stromelysin-3 matrix metalloproteinase. Cancer research.

[74] Wu E, Mari BP, Wang F, Anderson IC, Sunday ME, Shipp MA (2001). Stromelysin-3 suppresses tumor cell apoptosis in a murine model. Journal of Cellular Biochemistry.

[75] Murphy G, Nagase H (2008). Progress in matrix metalloproteinase research. Mol Aspects Med.

[76] Sun J (2010). Matrix metalloproteinases and tissue inhibitor of metalloproteinases are essential for the inflammatory response in cancer cells. J Signal Transduct.

[77] Vincenti MP (2001). The matrix metalloproteinase (MMP) and tissue inhibitor of metalloproteinase (TIMP) genes. Transcriptional and posttranscriptional regulation, signal transduction and cell-type-specific expression. Methods in molecular biology (Clifton, NJ).

[78] Geng JS, Song HT, Wang WR (2004). [Diversity of invasiveness and matrix metalloproteinases expression profile of human gastric carcinoma xenografted in different tissue environments]. Zhonghua bing li xue za zhi Chinese journal of pathology.

[79] Mosmann T (1983). Rapid colorimetric assay for cellular growth and survival: application to proliferation and cytotoxicity assays. Journal of immunological methods.

